# *“In general people aren’t excited about the vaccine…”*: Frontline perspectives on COVID-19 vaccine hesitancy across Syria

**DOI:** 10.1080/21645515.2023.2235239

**Published:** 2023-07-25

**Authors:** Mervat Alhaffar, Yazan Douedari, Natasha Howard

**Affiliations:** a Syria Research Group (SyRG), The London School of Hygiene & Tropical Medicine, National University of Singapore Saw Swee Hock School of Public Health; b Department of Global Health & Development, London School of Hygiene & Tropical Medicine, London, UK; c Free Aleppo University, Aleppo, Syria; d Saw Swee Hock School of Public Health, National University of Singapore and National University Health System, Singapore

**Keywords:** SARS-CoV-2, COVID-19, vaccine hesitancy, remote research, Syria, conflict

## Abstract

COVID-19 vaccine hesitancy is a new phenomenon in Syria, about which relatively little is known. We aimed to explore this, drawing from 37 semi-structured interviews with frontline health-workers and service-users across Syria’s major military areas-of-control. We found COVID-19 vaccine hesitancy was common and increasing among service-users and less common, but still present, among health-workers in all areas. Interrelated reasons included pragmatic fears of novel vaccine risks, unreliable information, and conflict-related hesitancies as a form of resistance or reasserting some perceived control, particularly outside Al-Assad government-controlled areas. Vaccine hesitancy has thus become a socio-political issue, requiring macro-level responses, across Syria.

## Introduction

### Vaccine hesitancy in conflict-affected settings

Vaccine hesitancy is a growing global phenomenon,^1^ made prominent by COVID-19.^2,3^ SAGE defines vaccine hesitancy as “*a delay in acceptance or refusal of vaccination despite availability of vaccination services*”^4^ and recognizes it as complex and context specific, potentially affected by misinformation, health inequalities, socioeconomic disadvantages, violence, and inaccessibility.^1^ Peretti-Watel *et al* reflected on the ambiguous and heterogeneous nature of vaccine hesitancy as “a kind of decision-making process”,^5,6^ though hesitancy remains largely attributed to superficial assumptions of ‘poor awareness’ rather than alternative prioritizations.

Conflicts involve violence, insecurity, and protracted disruptions to infrastructure and services, including vaccination and information.^7^ People’s lived realities during conflict may be significantly removed from rapid global vaccine development and deployment, with immediate survival often prioritized over potential vaccine-preventable infections.^8^ Thus, vaccination perceptions and engagement can be negatively affected.^2^ Perceptions of healthcare quality during conflict can also worsen vaccine hesitancy, sometimes due to mistrust of authorities including health authorities.^3^ The COVID-19 pandemic increased awareness of vaccination globally, though coverage rates remain low in many countries, affecting health authorities’ abilities to control disease.^9^ Emergence of new variants is particularly risky in conflict-affected settings, given inadequate health-workers and resources, indicating their importance to disease control globally.^2^ Conflict dynamics likely further complicate vaccine hesitancy but relatively little research has examined this.

### Syria’s vaccine hesitancy

Our research in Syria highlighted significant COVID-19 vaccine hesitancy in the three main areas of military control, i.e. opposition-controlled (OCA) northwest, autonomous administration-controlled (AACA) northeast, and Al-Assad government-controlled (GCA) central-south areas.^10^ Understanding vaccine hesitancy in Syria requires contextual consideration. After more than a decade of destructive multiparty conflict, Syria’s population is overburdened by socioeconomic and educational difficulties alongside a shattered health system.^8^ COVID-19 vaccination began 25 February 2021 in Damascus (GCA) through the COVID-19 Vaccines Global Access (COVAX). Initial COVAX vaccines were only sufficient to vaccinate 3% of the population, selected by Damascus Ministry of Health for GCA/AACA and Syria Immunisation Group (SIG) for OCA, which was the standard proportion for COVAX-eligible countries but interpreted by many Syrians as inequitable.^11–13^ By 5 May 2021, the Damascus Ministry of Health (MoH) developed an online platform for people in GCA and AACA to register for vaccination, raising fears the platform would be used to track dissidents. Lack of public information about numbers and sources of COVID-19 vaccines elevated distrust, with Shibani et al finding only 37% of participants very likely to be vaccinated if vaccines were available, 37% believing misinformation about vaccination, 50% relying on social media for information, and 42% uncertain about COVID-19 vaccine formulation.^12^ Mohamad et al found fear of side effects was the main reason for hesitancy, with men more willing than women to be vaccinated due to readier access to reliable information.^14^

As part of a broader longitudinal study of COVID-19 response governance in Syria, we aimed to explore emerging vaccine hesitancy.

## Methods

Findings draw on vaccine-related responses, collected during three rounds of semi-structured interviews conducted in colloquial Syrian Arabic between April and September 2021, including 15 frontline health-workers and 22 service-users across the three main areas of military control in Syria (Table 1). We transcribed and analyzed data in Arabic using reflexive thematic analysis.^15^

**Table 1.** Participant characteristics.

ID	Profession	Area	Age group	Sex
GCA_F14	Doctor	GCA	20–30	Female
GCA_F19	Pharmacist	GCA	20–30	Female
GCA_F20	Medical student	GCA	20–30	Female
GCA_F17	Nurse	GCA	30–40	Female
OCA_F15	Midwife	OCA	50–60	Female
AACA_F16	Nurse	AACA	30–40	Female
AACA_F18	Nurse	AACA	20–30	Female
R5-O1	Doctor	OCA	40–50	Male
R5-O2	Midwife	OCA	30–40	Female
R5-O3	Doctor	OCA	30–40	Male
R5-G3	Dentist	GCA	30–40	Male
R5-G4	Doctor	GCA	30–40	Female
R5-A1	Doctor	AACA	30–40	Male
R5-A2	Doctor	AACA	50–60	Male
R5-A3	Nurse	AACA	20–30	Male
SU1	Mediaperson	OCA	18–25	Female
SU2	Educater	OCA	41–45	Female
SU3	Educater	OCA	31–35	Female
SU4	Educater	OCA	31–35	Female
SU5	Academic	AACA	41–45	Male
SU6	Engineer	AACA	31–35	Female
SU7	NGO staff	OCA	36–40	Male
SU8	Student	OCA	31–35	Male
SU9	NGO staff	OCA	36–40	Male
SU10	NGO staff	AACA	31–35	Male
SU11	Clerk	AACA	36–40	Male
SU12	NGO staff	AACA	41–45	Male
SU13	Clerk	AACA	26–30	Female
SU14	Lawyer	GCA	31–35	Female
SU15	Homemaker	GCA	41–45	Female
SU16	Unemployed	GCA	18–25	Female
SU17	Homemaker	GCA	41–45	Female
SU18	Retired	GCA	61–65	Male
SU19	Homemaker	GCA	66–70	Female
SU20	Graphic designer	GCA	18–25	Female
SU21	Businessman	GCA	36–40	Male
SU22	Lawyer	GCA	26–30	Male

## Findings

We found COVID-19 vaccine hesitancy was common and growing among service-users and less common, but still present, among health-workers. Major overlapping themes were: (i) pragmatic fears of novel vaccine risks; (ii) context of unreliable information; and (iii) conflict-related hesitancy as resistance.

### Pragmatic fears of novel vaccine risks

Safety and efficacy concerns were common among all service-users in all areas of military control, either expressed generally for all COVID-19 vaccines, due to perceived absence of long-term testing, or specific to certain brands. Thus, most hesitancy expressed a pragmatic fear of the unknown along with distrust in the development process.

The widespread perception in the community is that the vaccine is unknown, and its benefits are unknown R5O2

I hesitate to take it. I feel it appeared suddenly and it’s not tested R5R1

These hesitancies gained strength as COVID-19 became relatively familiar and less frightening. Fears among most participants shifted from the disease to the vaccine because it was newer, required an active choice to get vaccinated, and COVID-19 appeared survivable.

I wouldn’t take it because the vaccine is still under development. And myself I got infected with COVID-19. I only felt like severe cold symptoms and then I recovered, but I didn’t feel it was very dangerous for me. So, I think may be useful for a small group of people but not for me SU8^8^

Our immunity is good and unless it was tested on everyone and proven safe, I would never take it or have anybody in my family take it SU17^8^

Several expressed concerns about the multiplicity of brands (an outcome of bilateral vaccine donations and COVAX brand neutrality).

The problem is that this vaccine has different names [brands] while the polio vaccine is one type for everyone, right? How come COVID vaccine has different names and types? This variety causes fear, right? It means that there is no trust in any vaccine, if there were trust in one vaccine allocated for COVID, all people should get vaccinated with it OCA_F15

### Context of unreliable information

Vaccine concerns appeared heightened by an overload of relatively unreliable information and the absence of a clear trusted source, as most people relied on social media sources, particularly Facebook and WhatsApp. Misinformation (i.e. ‘false information that is spread, regardless of intent to mislead’) sharing was common among all service-users in all areas of military control.

I did not take the vaccine, to be honest, I don’t know where it is coming from. Trust [in authorities] is missing, there is no credibility R5S1

A popular misinformation narrative, reported by most service-users, was that people infected with SARS-CoV-2 developed long-lasting immunity and thus did not need vaccination.

Some people who got COVID-19 are saying they have immunity now R5S3

Rumors and unsubstantiated theories were widely shared across Syria, linked by most participants to poor public trust in governing bodies in all three areas. For example, participants’ worries about the AstraZeneca vaccine’s (i.e. Covishield or Vaxzevria) reputation for blood clots, were worsened by rumors of Syrians having died from vaccination.

[Local authorities] stopped AstraZeneca because two people took it and died R5R1

Some people spread rumors that one might die [after vaccination] R5O2

While it was not possible to determine whether disinformation (i.e. ‘deliberately biased or false information that is intended to mislead’) was being spread, false information was also shared among some health professionals.

I was excited to get the vaccine [… but] a small percentage of doctors and nurses were saying it causes fetal malformations in women […], or that we may die after two years [of being vaccinated] GCA_F17

Some participants related widespread false information and distrust to poor community engagement and vaccine-related information provision by governing bodies in all three areas of control, particularly GCA.

There is no awareness raising in the media that people should take the vaccine R5G2

There’s no government official with the awareness to provide us with accurate information SU3^8^

### Conflict-related hesitancy as resistance

Conflict-related vaccine hesitancy was common among both service-users and health-workers, particularly outside Al-Assad government-controlled areas. These hesitancies related to fears expressed by most OCA and AACA participants that Russia and the Al-Assad government were using COVID-19 vaccines as weapons of war against populations in areas outside its military control and thus took the form of resistance to anything offered by the Al-Assad regime or its allies. This should be understood within the context of ongoing weaponization of healthcare in Syria and bombardment of civilians by the Al-Assad government and its allies in both areas.^16,17^ For example, during the 11-year conflict, over 542 attacks on health facilities and 831 health-worker killings by the Al-Assad government and Russian forces were documented.^18,19^ Al-Assad’s government further weaponized healthcare by withdrawing or blocking health services including vaccination,^16,17^ thus experience influenced distrust.

In AACA, some health-worker concerns about COVID-19 vaccine safety coalesced around perceived inadequacies of the national (Al-Assad government-controlled) vaccination programme and the cold-chain through which vaccines coming to the northeast were channeled despite ongoing conflict.

Some doctors said the vaccine is offered through the [Assad] regime and the regime killed 1.5 million, so how would I take the vaccine? A regime soldier could simply disconnect the vaccines refrigerator. A good percentage of doctors and educated people refused the vaccine because it’s channeled through the regime R5A3

In OCA, most participants’ concerns were phrased in relation to the perceived ineffectiveness or risk of taking the Russian-produced Sputnik vaccine that was most readily available in northwest Syria. Sinopharm and Sputnik vaccines were resisted due both to the poor perceived reputations of Russian and Chinese products and, in several cases, because these countries supported the Al-Assad government (e.g. in addition to Russia’s military support, Syria was among its top three bilateral vaccine donation recipients.^13^

In general people aren’t excited about the vaccine. Rumours say that Russia will send the vaccine to kill us all SU2^8^

The point is, the regime used chemical attacks, bombarded us with planes and all sorts of weapons and nobody cared how Syrians were killed before, now they’re concerned [about us] because of COVID-19 NW_F10

## Discussion

COVID-19 vaccine hesitancy is a global phenomenon that also affects Syria, despite previous high uptake of routine vaccination.^20^ Identifying the main issues affecting ongoing COVID-19 vaccine hesitancy in Syria has ramifications for routine vaccination and value for future epidemics. Our findings support and help explain survey findings from both Mohamad et al and Shibani et al.^12,14^ They also add contextual nuance to vaccine hesitancy as experienced in conflict-affected settings, where fears for survival and distrust of authorities can amplify the power of misinformation and disinformation.^21^ Siddiqui et al, in a narrative review of increased vaccine hesitancy in conflict-affected settings found similarly that major reasons for vaccine hesitancy included conflict severity and instability in Yemen, misinformation in Afghanistan and Palestine, and fears about vaccine safety and side effects in Palestine and Yemen.^3^

While many countries have struggled with COVID-19 vaccine hesitancy, much of it related to fears of COVID-19 vaccines being new and ‘untested’, what was particularly relevant in Syria was the seemingly pervasive assumption that nobody cared about Syrians’ suffering during the conflict, which reinforced doubt about the value of international COVID-19 responses. We noted latent themes of vaccines as riskier than COVID-19 and hesitancy as resistance. The first related to the ways illnesses reported or experienced reduced COVID-19’s importance in comparison to urgent livelihood and security concerns. The latter could serve both as means of re/exerting control in an uncontrollable environment and as resistance against the Syrian government and allies, resonating unexpectedly with US socio-politics of vaccine hesitancy as noted by Sorrell & Butler and others^22^.

Given the protracted multiparty conflict, the major issues of fear and distrust, particularly of the Al-Assad government, must be considered by vaccination partners such as Gavi the Vaccine Alliance, UNICEF, and WHO in a culturally and contextually sensitive manner that accounts for the lived realities of Syrians in different areas-of-control. For example, COVAX partner assumptions that vaccines should be provided to AACA by Damascus MoH ignored conflict-related sensitivities, with hesitancy potentially expressing survival fears and resistance. Channelling vaccines through local health authorities in each area of military control rather than crossline through the Al-Assad government would likely increase trust and vaccine uptake. Similarly, provision of Sputnik V in OCA and AACA, with its negative associations with Russian bombing, will likely reduce its uptake. In OCA, leveraging pro-vaccine norms enhanced by SIG’s ongoing work could help mitigate hesitancy.

While improving individual vaccine awareness and understanding may improve uptake somewhat, combining this with coordinated local authority and vaccination partner actions to improve trust and mitigate socio-political factors would likely do more. Using tools such as the vaccine hesitancy matrix, to consider contextual, group, individual, and vaccine-specific influences of behavioral decisions, could help planning and implementation.^4^

Public engagement could be improved by local authorities in all areas of control. Social media, particularly Facebook, is popular across Syria and could be used to counter misinformation and disinformation, while encouraging ongoing public dialogue about vaccination. Community leaders and influencers can share positive vaccination experiences, as has been done in countries with successful responses. Local health authorities should actively work to increase public trust, for example by initiating transparent communication about vaccination and other health issues. A holistic approach, mitigating socio-political issues combined with accurate information widely disseminated by trusted sources (e.g. WHO), would thus help address issues underlying vaccine hesitancy in Syria.
